# Prevalence of overweight/obesity and its association with fast food consumption among adolescents in Southern Ethiopia, 2022: a community-based cross-sectional study

**DOI:** 10.3389/fnut.2024.1475116

**Published:** 2025-01-27

**Authors:** Chernet Elias, Awoke Abraham, Chernet Asrat, Tagese Yakob, Daniel Girma

**Affiliations:** ^1^School of Public Health, College of Health Science and Medicine, Wolaita Sodo University, Sodo, Ethiopia; ^2^Department of Human Nutrition, Wolaita Zone Health Office, Sodo, Ethiopia; ^3^Data Quality Office, Nifas Silk Lafto Worda 11, Addis Ababa, Ethiopia

**Keywords:** fast food, overweight/obesity, prevalence, adolescent, urban

## Abstract

Overweight and obesity are body mass index (BMI) for age *Z*-score > +1SD and > +2SD, respectively. Despite its largest contribution to overweight/obesity, there is little attention in our country about fast-food consumption. Hence, this study aimed to assess the prevalence of overweight/obesity and its association with fast-food consumption among adolescents in Southern Ethiopia. From 14 June 2022 to 15 July 2022, a cross-sectional study design was conducted using a structured questionnaire and anthropometric measurements, with a sample size of 563. The data were coded, cleaned, and imported into EPI-INF version 7.2 before being exported to SPSS version 25 for analysis. All variables with *p*-values less than or equal to 0.25 in bivariate logistic regression were candidate variables for multivariable logistic regression. The response rate of this study was 97%. Based on BMI, the combined prevalence of overweight/obesity was 6.0% (95% CI: 4.2–8.4%). Adolescents who are female, come from high-income families, and eat fast-food frequently are more likely to be overweight or obese. Future health initiatives aimed at lowering the prevalence of overweight and obesity in adolescents should pay special attention to female adolescents and those from high-income families.

## Introduction

Overweight and obesity are described by body mass index (BMI) for age *Z*-score > +1SD and > +2SD above the WHO Growth Reference median, respectively. Overweight is BMI ≥ 85th to <95th percentile for age and sex, and obesity is BMI ≥ 95th percentile for age and sex (1). Overweight/obesity among adolescents is dramatically increasing and becoming a global concern. In 2022, more than 390 million children and adolescents between the age of 5 and 19 years were overweight, with 160 million of them suffering from obesity (2). In addition, overweight and obesity are the main contributors to type 2 diabetes mellitus, insulin resistance, cardiovascular disease, and cancer-related consequences. Previously, overweight and obesity were an issue in high-income countries, but currently, it is expanding to low-income and middle-income countries, especially in metropolitan areas (3, 4). Ethiopia is also experiencing a spike in both overweight and obesity. Therefore, a combined pooled prevalence of overweight and obesity among children and adolescents in Ethiopia suggested 11.30% (95% CI: 8.71, 13.88), whereas the individual pooled prevalence was 8.92 and 2.39%, respectively (5). Recent research suggested that increased availability to fast-food restaurants and a higher intake of ultra-processed food might increase the prevalence of metabolic syndrome, particularly abdominal obesity and diabetes mellitus (6, 7). Several studies have also shown that increased fast-food consumption of adolescents is associated with a rise in overweight and obesity (8–10).

Even though the causes of obesity are thought to be numerous, fast food has been related to the issue in many nations due to its rising popularity, high-calorie content, and large serving sizes (11). A study in college students found that frequent fast-food consumption raised the prevalence of obesity from 3.6 to 35.4% for those who ate fast food less than four times per week and more than four times per week, respectively (12). Fast food is a mass-produced food designed for commercial resale, with a strong priority placed on service speed and consumption outside of the home (13). It is calorie-dense, lacks micronutrients, has a high glycemic index and low fiber content, and is served in large quantities (14). Additionally, it contains a lot of saturated and trans fatty acids (15).

People’s eating habits of people are changing in Ethiopia, with more urban dwellers preferring ready-to-eat (quick) foods accessible in open markets and low-cost eateries (16). Cheap, nutrient-poor, and energy-dense foods are currently simpler to find than nutritious foods. In metropolitan areas, the intake of processed meats, sweetened beverages, saturated fats, cholesterol, and sodium is increasing, according to a recent study (17).

A number of studies carried out abroad show an adverse association between fast food and overweight or obesity. In Indonesia, a study suggested both waist circumference and BMI have a significantly negative association with increased fast food consumption (18). Another study, in the UK, indicated participants who consumed fast food on a weekly basis had ~20% lower odds of being overweight than those who consumed it rarely or never (19).

One might expect that youth who are more educated would make better choices regarding nutrition; however, regardless of their level of education, adolescents are becoming increasingly overweight or obese. Studies in Ethiopia, however, have ignored young, preschoolers concentrating on adolescent students instead. In Ethiopia, the economy is growing fast, facing rural–urban migration problems. Due to this transition, the food habits of people are changing with more people in urban areas eating outside the home and giving preference to fast foods (16). Wolaita Sodo is one of the fastest growing cities in the country, with new urban region developments growing largely in all directions of the town (20).

Despite being the main cause of overweight and obesity, fast-food consumption has received little attention in our country. The goal of this study was to concentrate more on fast-food consumption as a primary covariate, in Sodo town, at the community level.

In addition, conducting this study at the community level enabled us to include both in-school and out-of-school adolescents. Moreover, investigating the prevalence of overweight/obesity and its associated factors is so essential to stimulating action before the onset of chronic disease. It can also serve as a piece of evidence for the development of national food policies, programs, and dietary guidelines as this is the first community-based study in a study area.

The hypothesis of the study is, ‘Adolescents who eat fast food frequently have a higher likelihood of being overweight or obese than those who eat fast food less frequently, in Sodo town.’

## Methods

### Study setting, design, and period

From the 14th of June up to the 15th of July 2022, a community-based cross-sectional study was carried out in Wolaita Sodo town, southern Ethiopia. The town has 6°54′ N latitude, 39°45′ E longitude, and an altitude of roughly 1900 m; it is situated 320 kilometers southwest of capital city of Ethiopia of Addis Ababa and 160 kilometers west of the regional town of Hawasa. The Federal Democratic Republic of Central Statistics Agency of Ethiopia reported in 2018 that the town had a total population of 132,271 (62,340 men and 69,931 women), of whom 3,263 were young people (aged 15–24 years) (21). According to recent demographic data in the administrative office of town, the town is divided into seven sub-cities, which have 46,956 households (HH) and a population of 286,566.

### Population

Adolescents in Wolaita Sodo town were the source population, whereas those in three selected subcities of Wolaita Sodo town were the study population. Individual adolescents from selected households were included in the study. However, adolescents who had a physical disability (unable to stand erect), mental disability, pregnancy, or edema due to some other pathological causes were excluded.

### Sample size determination and sampling procedure

Using a single population proportion formula, the sample size was calculated under the following assumptions: a 95% confidence level, a margin of error of 4%, a design effect of 1.5, and a non-response rate of 5%. We used the outcome variable overweight/obesity, which has a combined prevalence of 18.2% among school-aged adolescents in Addis Ababa, to calculate the size of our sample (22). Our final sample was 563.

Currently, there are seven (7) sub-cities in Wolaita Sodo town administration. Initially, three sub-cities (Wadu Amba, Fana Womba, and Mehal) were randomly selected, and the census was conducted to determine the total number of adolescents in each sub-city. During the process of the census, the households with one or more adolescents were given a new consecutive code, and the number of adolescents was recorded.

**Figure 1:**
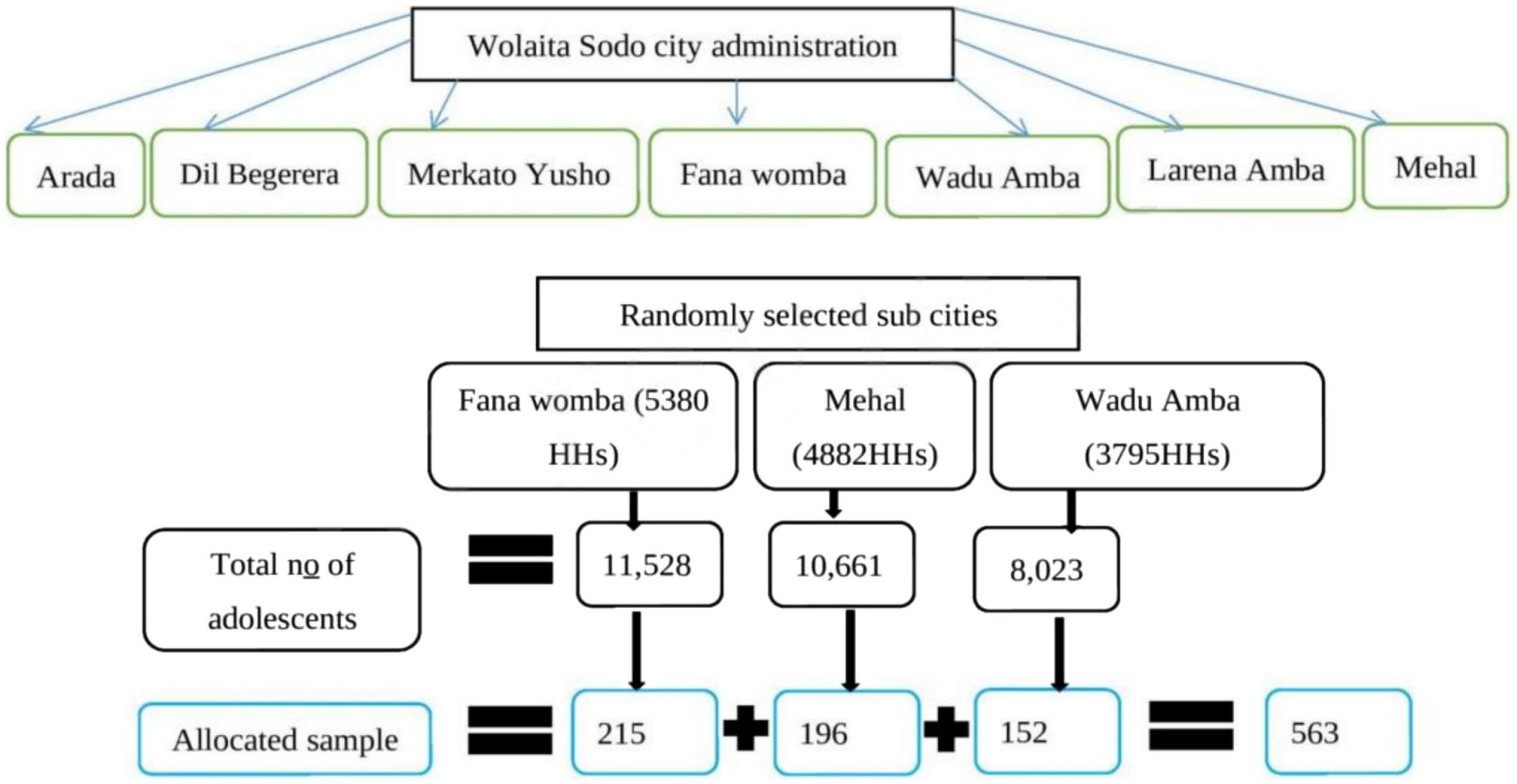
Sampling procedure and sample size allocation.

Using the new codes, labeling was done, and the total number of adolescents in each sub-city was calculated. Then, the total sample size was allocated to each sub-city proportionally to the total number of households with adolescents within the sub-city. Finally, study units were selected by the systematic random sampling method. The sampling procedure is shown in Figure 1.

### Study measures/variables

Overweight/obesity was the outcome variable, whereas socio-demographic variables, fast-food consumption, physical activity, alcohol intake, and sedentary lifestyle characteristics were the independent variables in this study.

### Operational definition

**Overweight:** BMI-for-age *z*-score is greater than +1 SD above the WHO growth reference median.

**Obesity:** BMI-for-age *z*-score is greater than +2 SD above the WHO growth reference media.

**Fast food: Foods** that are packaged, easily accessible from vending machines, simply prepared, and served quickly (such as pizza, burgers, chocolate, ice cream, cookies/cake, chips, soft drinks, and biscuits).

**Frequent fast-food consumers**: Those adolescents who took fast food two or more times in the last 1 week.

**Lack of cooking skills**: Lack of ability to perform tasks related to the preparation of food at home.

**Alcohol intake:** Intake of alcoholic beverages (beer, wine, whiskey, Areke, Tela, Tej, etc.) for a non-medicinal purpose within the last 1 month.

**Sometimes take alcohol:** Intake of alcoholic beverages less than or equal to one time a week.

**Rarely take alcohol:** Intake of alcoholic beverages less than or equal to one time a month.

### Data collection procedures

Data associated with socio-demographic variables, dietary information, habits of fast-food consumption, and the like were collected using face-to-face interviews using a questionnaire developed after a thorough review of literature. Obesity index data such as waist and hip circumferences for calculating waist–hip ratio (WHR), and height and weight for computing BMI, were collected. Waist and hip circumference were measured by an anthropometric tape meter; the height of subjects was measured by a standard meter stick; and the weight of study subjects was taken to the nearest 0.1 kg using a calibrated digital balance in standing position by trained health professionals.

Height was measured to the nearest 0.5 cm after participants removed their heavy clothing and shoes, and waist and hip circumference measurements were taken at the narrowest and widest areas, respectively. Data were collected on weekend days (Saturday and Sunday) in order to address school-going adolescents.

### Data quality assurance

Two days of training for data collectors and supervisors was given prior to the actual survey. Standardization was done among 10 adolescents before the actual survey and systematized based on the result of anthropometric measurements. All three data collectors were degree holders in public health. Three assistants (one for each data collector) were assigned. All six of them speak local as well as Amharic language. A pretest was conducted on 5% of the questionnaire in the Merkato Yusho sub-city, which was not included in actual data collection. Appropriate modifications were made after analyzing the pretest results before the actual data collection. The reliability test results obtained Cronbach’s alpha value of 0.89; then, the instrument was declared reliable.

### Data processing and analysis

Data were cleaned, coded, and entered into EPI-INFO version 7.2 before being exported and analyzed in SPSS version 25. Anthropometric data and other crucial variables were imported into WHO Anthro-plus software, which uses WHO 2007 population references to convert anthropometric data into body mass index for age z-score (BAZ) while taking age and sex into account. We calculated WHR using an open waist-to-hip ratio calculator and used the WHO cutoff points of 0.94 cm for men and 0.85 cm for women to determine central obesity. Frequency tables, proportions, and texts were used to present the descriptive statistics.

Pearson correlation was used to ascertain the correlation and strength of the association between consuming fast food and being overweight or obese. Bivariate logistic regression analyses were also conducted to evaluate the relationship between other independent variables and the dependent variable. In the multivariable logistic regression model, variables that exhibited an association (*p*-value of 0.25 in the bivariate analysis) were included. Outputs with *p*-values of ≤0.05 were deemed statistically significant. Before the data analysis, normality was checked for continuous variables using the Shapiro–Wilk normality test and visualized using Q-Q plots.

We found that they were derived from a population with a normal distribution (*p*-value >0.05). The Hosmer–Lemshow and variance inflation factor (VIF) tests were used to determine the model fitness and multicollinearity, respectively.

## Results

### Socio-demographic characteristics of the study subjects

A total of 546 adolescents participated in this study, with a response rate of 97%; 259 (47.4%) were early adolescents aged 10–14 years, and 287 (52.6%) were late adolescents aged 15–19 years. 325 (59.5%) of the respondents were females, and 221 (40.5%) were males. More than two-thirds 380 (69.6%) of them were protestant Christians, followed by Orthodox Tewahedo 140 (25.6%) and Muslim 20 (3.7%) and 64.7% had primary education (Table 1).

**Table 1:** Demographic and socioeconomic characteristics of adolescents in Wolaita Sodo town, Southern Ethiopia, 2022.

Variables	Category	Frequency (*n* = 546)	Percentage
Age	Early adolescents (10–14)	259	52.6
	Late adolescents (15–19)	287	47.4
Sex	Female	325	59.5
	Male	221	40.5
Marital status	Single	538	98.5
	Married	8	1.5
Religion	Protestant	380	69.6
	Orthodox	140	25.6
	Muslim	20	3.7
	Others*	6	1.1
Occupation	Students	527	96.5
	Merchants	10	1.8
	Daily workers	4	0.7
	Others**	5	0.9
Father’s occupation	Government employee	268	49.1
	Merchants	216	39.6
	Daily workers	37	6.8
	Others***	25	4.6
Mother’s occupation	Government employee	179	32.8
	Merchants	134	24.5
	Housewives	216	39.6
	Daily workers and others****	17	3.1
Education of participants	Primary	353	64.7
	Secondary	178	32.6
	College	15	2.7
Father’s education	No formal education	29	5.3
	Primary	130	23.8
	Secondary	162	29.7
	College and above	225	41.2
Mother’s education	No formal education	30	5.5
	Primary	153	28.0
	Secondary	164	30.0
	College and above	199	36.4
Household monthly income (in ETB)	Less than 3,000	137	25.1
	3,001–5,000	112	20.5
	5,001–10,000	170	31.1
	More than 10,000	127	23.3

*Catholic, Adventist; **domestic worker, housewife; ***farmer, pastor; ****pastor, unknown.

### Habits and patterns of fast-food consumption of the respondents

Regarding fast-food consumption, 176 (32.2%) of the respondents were frequent (>2 times) consumers, of which 103 (58.5%) were females and 73 (41.5% were males); 130 (23.8%) of the respondents did not eat any kind of fast food in the past 7 days, and the remaining 416 (76.2%) ate fast food at least one time in the past 7 days. Of the 416 who ate fast food at least one time a week, approximately 115 (27.9%) consumed it from home, restaurants, and streets, 90 (21.6%) consumed it from restaurants and streets, and 77 (18.5%) ate only from their home. Most of the fast-food consumers, 132 (31.7%), preferred it because of its tasty nature, cheap price, recreational purpose, and convincing advertisements, followed by 102 (24.5%), who preferred it because of its tasty nature and convincing advertisements only (Table 2).

**Table 2:** Habits and patterns of fast-food consumption among adolescents in Wolaita Sodo town, Southern Ethiopia, 2022.

Fast-food characteristics	Category	Frequency	Percentage
Fast-food consumption	≤2 times per week	370	67.8
	>2 times per week	176	32.2
Fast-food consumption by sex	Male	73	41.5
	Female	103	58.5
Place to get fast food	Home only	77	18.5
	Home and restaurant	62	14.9
	Home and street	72	17.3
	Home, restaurant, and street	115	27.6
	Restaurant and street	90	21.6
Reasons for preferring fast foods	Easily accessible, cheap, and recreational purpose	76	18.3
	Tasty, recreational purpose, and advertisement	60	14.4
	Tasty and cheap	46	11
	Tasty and advertisement	102	24.5
	Tasty, cheap, recreation purpose and advertisement	132	31.7

### Dietary habits and meal patterns of adolescents

Three hundred twenty-five (59.5%) of the respondents said that they used fruits two to four times in the past 7 days, and 81 (14.8%) used them once per day. Regarding the intake of vegetables, 218 (39.9%) ate the vegetables two to four times in the past 1 week, followed by 129 (23.6%) who used more than two times per day. One hundred and eighty (33%) ate bread and cereals two to four times per week; 117 (21.4%) used them once a day, and 100 (18.3) ate more than two times per day. Regarding the animal source foods, 188 (34.4%), 133 (24.4%), 123 (22.5%), and 80 (14.7%) of the respondents ate meat and eggs one time a week, two to four times per week, less than once a week, and once per day, respectively. The majority of the adolescents (474) (86.8%) consumed snacks, with 201 (42.4%) taking them sometimes, 170 (35.6%) were daily users, and 103 (21.7%) use them rarely (Table 3).

**Table 3:** Dietary habits and meal patterns of adolescents in Wolaita Sodo town, Southern Ethiopia, 2022.

Variables	Category	Frequency	Percentage
Frequency of intake of fruits in the past 7 days	Less than once a week	18	3.3
	Once a week	91	16.7
	Once per day	81	14.8
	Two to four times a week	325	59.5
	More than two times per day	31	5.7
Frequency of intake of vegetables in the past 7 days	Less than once a week	47	8.6
	Once a week	54	9.9
	Once per day	98	17.9
	Two to four times a week	218	39.9
	More than two times per day	129	23.6
Frequency of intake of bread and cereals in the past 7 days	Less than once a week	98	17.9
	Once a week	51	9.3
	Once per day	117	21.4
	Two to four times a week	180	33
	More than two times per day	100	18.8
Frequency of intake of meat and eggs in the past 7 days	Less than once a week	123	22.5
	Once a week	188	34.4
	Once per day	80	14.7
	Two to four times a week	133	24.4
	More than two times per day	22	4
Frequency of intake of milk, cheese, and yogurt in the past 7 days	Less than once a week	54	9.9
	Once a week	166	30.4
	Once per day	144	26.4
	Two to four times a week	130	23.8
	More than two times per day	52	9.5
Frequency of intake of sugar and sweets in the past 7 days	Less than once a week	25	4.6
	Once a week	67	12.3
	Once per day	133	24.4
	Two to four times a week	127	23.3
	More than two times per day	194	35.5
Snack	Yes	474	86.8
	No	72	13.2
Frequency of snacks	Daily	170	35.9
	Sometimes	201	42.4
	Rarely	103	21.7

### Physical activity, alcohol intake, and sedentary lifestyle

The participants’ physical activity statuses related to sport and recreation revealed that only 23 (4.2%) participated in vigorous (high intensity) physical activity, whereas the majority (409, 74.9%) participated in moderate physical activity, and 114 (20.9%) of the participants had only low-intensity physical activity. Regarding the alcohol intake of the respondents, nearly all (530 [97.1%]) had no habits of taking any kind of alcoholic beverages. Moreover, the remaining 16 (2.9%) had habits of taking alcohol, of which seven (7) consumed sometimes and nine (9) took rarely. The sedentary behavior of the respondents showed that 269 (49.3%) of the adolescents spent watching TV programs, playing video games, or browsing the internet less than or equal to 120 min a day, whereas 277 (50.7%) spent more than 120 min a day. Regarding sleep duration, 94 (17.2%) spent short (< 8 h), 407 (74.5%) spent normal (8–10 h), and 45 (8.2%) spent long (>10 h) sleeping (Table 4).

**Table 4:** Physical activity, alcohol intake, and sedentary behavior of adolescents in Wolaita Sodo town, southern Ethiopia, 2022.

Variables	Category	Frequency	Percentage
Physical activity	Low physical activity	114	20.9
	Moderate physical activity	409	74.9
	High physical activity	23	4.2
Alcohol intake	Yes	16	2.9
	No	530	97.1
Times spent by watching TV, playing video games, or browsing the internet	≤120 min	269	49.3
	>120 min	277	50.7
Sleep duration (within 24 h)	Short (< 8 h)	94	17.2
	Normal (8–10 h)	407	74.5
	Long (>10 h)	45	8.2

### The prevalence of overweight/obesity

**Figure 2:**
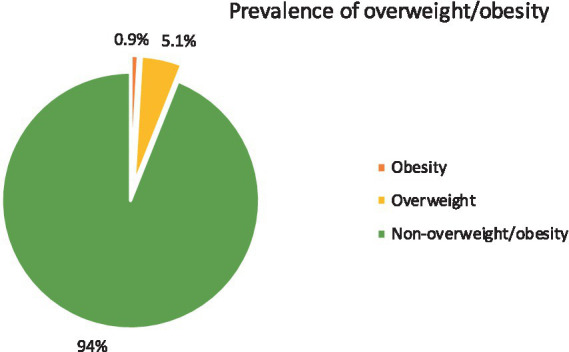
Prevalence of overweight/obesity.

The combined prevalence of overweight/obesity was 6.0% (95% CI: 4.2–8.4%) based on BMI-for-age categorization. The separated prevalence of overweight and obesity was 5.1% (95% CI: 3.4–7.3%) and 0.9% (95% CI: 0.3–2.1%), respectively. In addition, the prevalence of overweight/obesity was 7.9% based on WHR. Overweight/obesity was found to be 4.7% among females and 1.3% among males, respectively, and 4.2% among late adolescents and 1.8% among early adolescents. The prevalence of overweight/obesity is 10.2% among frequent (>2 times) fast-food eaters and 4.1% among non-frequent consumers (Figure 2). There is a substantial correlation between the frequency of fast-food consumption and overweight/obesity.

The frequency of fast-food consumption and BMI-for-age *Z*-score level was shown to be a statistically significant correlation (*p* < 0.01), with a moderately strong positive association (Pearson *r* = 0.56) between the frequency of fast-food consumption and BMI-for-age *Z*-score (as shown in Table 5).

**Table 5:** The linear correlation between BAZ and frequency of fast-food consumption among adolescents in Wolaita Sodo town, Southern Ethiopia, 2022.

	Correlations	BMI-for-age *Z*-score	Frequency of fast food consumption
BMI-for-age *Z*-score	Pearson correlation	1	0.561**
	Sig. (two-tailed)		0
	*N*	546	363
Frequency of fast-food consumption	Pearson correlation	0.561**	1
	Sig. (two-tailed)	0	
	*N*	363	546

^**^Correlation is significant at the 0.01 level (two-tailed).

Sex of participants, age group of the respondents, mother’s occupation, household monthly income, frequency of fast-food consumption, frequency of meat and egg consumption, and physical activity level were variables having a *p*-value of less than 0.25 in bivariate analysis. Therefore, these variables were fitted in the multivariate logistic regression to get the adjusted effect of each covariate. After adjusting, the sex of participants, household monthly income, and frequency of fast-food consumption were significantly associated with overweight/obesity at a 95% confidence level and a *p*-value of ≤0.05.

Adolescent girls were 1.75 times more at risk of becoming overweight and obese than adolescent males (AOR = 1.75 [95%CI: 1.03–3.40]). Adolescents from households with monthly incomes greater than 10,000 EBT were 3.2 times at higher risk for becoming overweight or obese as compared to adolescents whose family income was less than 3,000 EBT per month (AOR = 3.21 [95%CI: 1.22–5.42]). Prevalence for overweight/obesity was almost three times higher for those who frequently consumed fast food, than those adolescents consuming fast foods two times or less than two times within a week (AOR = 2.98 [95%CI: 1.89–4.76]) (Table 6).

**Table 6:** Odds ratios of overweight/obesity for significantly associated factors (logistic regression analysis) for adolescents in Wolaita Sodo town, Southern Ethiopia, 2022.

Variables	Categories	Non-overweight/obesity (%)	Overweight/obesity (%)	Crude OR[95%CI]	*p*-value s	AdjustedOR [95% CI]	*p*-values
Sex*	Male	214 [96.8]	7 [3.2]	1		1	
	Female	299 [92.0]	26 [8.0]	2.65 [1.11–6.23]	0.04	1.75 [1.03–3.40]	0.01
Age group	Early adolescents	249 [96.1]	10 [3.9]	1		1	
	Late adolescents	264 [92]	23 [8]	2.1.7 [1.01–14.43]	0.12	2.48 [1.22–18.56]	0.12
Mother’s occupation	Government employee	171 [95.5]	8 [4.5]	1		1	
	Merchants	8 [4.5]	11 [8.2]	1.89 [1.00–15.34]	0.177	0.87 [0.32–2.37]	0.26
	Housewives	207 [95.8]	9 [4.2]	0.89 [0.32–0.98]	0.883	8.95 [2.06–38.87]	0.78
	Daily workers and others	12 [70.6]	5 [29.4]	8.90 [2.51–34.23]	0.001	2.48 [1.08–5.70]	0.31
Household monthly income (in ETB)*	Less than 3,000	132 [96.3]	5 [3.7]	1		1	
	3,001-5,000	105 [93.8]	7 [6.2]	1.76 [1.34–3.14]	0.2	2.05 [1.22–3.11]	0.04
	5,001-10,000	158 [93]	12 [7.0]	2.00 [1.01–4.10]	0.23	1.42 [1.02–2.10]	0.09
	More than 10,000	118 [93]	9 [7.0]	2.02 [1.14–5.12]	0.08	3.21 [1.22–5.42]	0.01
Frequency of meat and egg consumption	Less than once a week	118 [95.9]	5 [4.1]	1		1	
	Once a week	179 [95.2]	9 [4.8]	1.20 [1.0 1–7.02]	0.16	1.47 [0.39–5.94]	0.56
	Once per day	75 [93.8]	5 [6.2]	1.57 [1.1 0–5.73]	0.22	1.64 [1.04–6.73]	0.48
	Two to four times a week	124 [93.2]	9 [6.8]	1.71 [2.1 2–8.31]	0.27	0.73 [0.20–0.92]	0.63
	More than two times per day	17 [77.3]	5 [22.7]	6.94 [2.1 2–21.10]	0.02	6.01 [1.27–28.46]	0.24
Fast-food consumption*	≤2 times per week	355 [96]	15 [4]	1		1	
	>2 times per week	158 [89.7]	18 [10.3]	2.69 [1.3 2–5.48]	0.001	2.98 [1.89–4.76]	0.001
Physical activity level	Low physical activity	103 [90.4]	11 [9.6]	1		1	
	Moderate physical Activity	389 [95.1]	20 [4.9]	0.48 [0.2 1–0.64]	0.24	0.43 [0,19–0.99]	0.48
	High physical activity	21 [91.3]	2 [8.7]	0.89 [0.4 2–0.96]	0.02	1.29 [0.23–7.08]	0.76

^*^Significantly associated variables in multivariate analysis.

## Discussion

Based on BMI-for-age classification, the combined prevalence of overweight/obesity was 6.0% (95% CI: 4.2–8.4%), and a statistically significant association (*p* < 0.01) was found between the frequency of fast-food consumption and BMI for the age *Z*-score level, with a positive correlation and moderate strength, with a Pearson’s *r* value of 0.56. The prevalence of overweight/obesity in our study is consistent with previous findings in the same location 5 years ago, which is 5.1% (23). However, the prevalence of obesity alone (0.9%) is higher than the previous finding, which is 0.3%. This could be because of a shift in lifestyle that involves less physical exercise, the impact of technology, intermittent economic volatility, and the adoption of Western diets (fast foods); and a difference in methods as this study was community-based.

However, the magnitude of overweight/obesity in this study is lower than results from South Africa, which was 16.9% (24), and Ghana and Uganda, where the prevalence of overweight/obesity was 15.9% (25). It is lower than the findings from South Wollo, which revealed the magnitude of overweight/obesity as 8.2% (26), and Bahr Dar town, which is 7.7 and 10.7% for overweight and obesity, respectively (27).

The socioeconomic background of the research population may be the cause of this; in Africa, being overweight is a sign of wealth, and the prevalence may vary depending on the economic standing of community.

The findings revealed a considerable sex difference in overweight (males: 0.9%; females: 4.2%) and obesity (males: 0.4%; females: 0.5%). Moreover, female adolescents were 1.75 times more likely to be overweight or obese than males. According to a Nepalese study, female adolescents were more likely to become overweight than male adolescents (28). This study is aligned with a study from Mekele, which suggested similar results; the odds of overweight and obesity among females were two times higher as compared to males (29). This finding also agrees with the result from meta-analysis, which showed that female adolescents were 3.23 times more likely to be overweight/obese than their male counterparts (5). This is again in line with findings from Hawasa town, in which girls were 5.14 times more likely to be overweight or obese compared with boys (30). Similar results were documented in studies from Dessie Town (26) and Dire Dawa (31). This discrepancy may result from women engaging in less physical activity than men, spending more time at home, and having more limited mobility as a result of cultural factors. It is also highly likely to be associated with general physiological differences between the sexes. In Ethiopia, due to some cultural constraints, girls were spending their free time doing sedentary things such as sitting and watching TV shows and movies, as well as assisting their mothers with home chores, whereas boys could have the opportunity to participate in outdoor games (32).

In this study, household monthly income had a statistically significant relationship with overweight and obesity. Adolescents from families with a monthly income of more than 10,000 ETB were 3.2 times more likely to become overweight/obese than adolescents from families with a monthly income of less than 3,000 ETB. Several reports revealed consistent findings. A study in Morocco found that the prevalence of overweight and obesity increases with higher household wealth (33). Meta-analysis in Ethiopia suggests adolescents from high-income families were 3.16 times more likely to be overweight/obese as compared to those from middle and low-income families (5). A substantial relationship between household income and overweight/obesity was also found in the study from Bahir Dar; adolescents from higher income families had a higher likelihood of being overweight/obese than those from lower income families (27).

In addition to this, a study from Addis Ababa reported that adolescents whose family’s income was more than 22,727 ETB per month were 4.1 times more likely to be overweight/obese (AOR = 4.1, 95% CI: 1.1, 15.8) than those families’ incomes less than or equal to 1,864 ETB per month (22) and a study from Hawasa town also showed a similar association (30). This may be due to the Western-style eating habits of adolescents from higher socioeconomic groups, which consume more high-fat, high-calorie foods in place of the wholesome, traditional diet of cereals, fruit, and vegetables.

In addition, they are even more exposed to a sedentary way of life. However, a cohort study from Taipei showed that children from low-income households had a significantly higher risk of becoming overweight/obesity than those from middle-and high-income households (34). Another qualitative study, from Hong Kong, reveals that adolescents from low-income families are more likely to engage in unhealthy dietary behaviors and, as a result, are more vulnerable to diet-related health problems (35). One possible explanation for this could be that adolescents from industrialized countries, while likely more aware of healthy diets, consume fast food because it is cheap and convenient.

About 32.2% of the respondents were frequent (>2 times) fast-food consumers, of which 105 (59%) were females and 73 (41% were males). This result is lower than the findings from Bangladesh (36) and Sri Lanka (37). Moreover, this finding is even lower than reports from Saudi Arabia (38). This could be related to differences in socioeconomic status, lifestyle, and easy access to junk and processed foods among the research population. According to one Italian survey, over 80% of kids have visited a fast-food restaurant at least once in their lives, yet just 15.5% of the sample said they frequented the establishment more than once a week (39). This is significantly less than what we found. Moreover, this might be because of a government plan that includes a number of programs designed to encourage pupils in that particular area to consume more fruits and veggies. A study in Saudi Arabia identified there was no significant relationship between BMI categories and father’s education level and mother’s education. However, the same study results show that while the fathers of most of the overweight and obese children were employed, the mothers of most of the overweight and obese children were not employed (40). However, our research showed no significant association between adolescents’ overweight/obesity and their parents’ career or level of education. This discrepancy may be due to the difference in type of study design and sample size.

In terms of frequency of fast-food intake, frequent fast-food users were almost three times more overweight/obese than those who consumed fast foods two times or less than two times per week, which is similar to data from Sri Lanka (37) and Saudi Arabia (38).

Another recent study, in the UK, found that increased visits to fast-food outlets were associated with higher BMI SD scores in adolescents (41), which is again in line with these findings. This might be due to the fact that fast foods are energy dense and high calories. However, there is one contrary finding from Hawasa town, among the adult community, in which no significant associations were found for frequency of consumption of fast foods and overweight/obesity (42). This may be due to differences in the study population and study period. Compared to states in the European Union, where the prevalence varies from 12% in the Netherlands to 36% in Malta, the prevalence of overweight and obesity in our study is significantly lower (43). This could be due to adolescents in developing nations still adjusting to a Western-style diet.

### Recommendation

WHO published the six implementation plans for the prevention and interventions of childhood and adolescents’ obesity in 2017. First, there is a need to implement comprehensive programs that promote the intake of healthy foods and reduce the intake of unhealthy foods and sugary beverages by children and adolescents. Second, comprehensive programs must promote physical activity and reduce sedentary behaviors in children and adolescents. Third, the program must integrate and strengthen guidance for non-communicable disease prevention with current guidance for preconception and antenatal care to reduce the risk of childhood obesity. Fourth, it is imperative to provide guidance on and support for healthy diet, sleep, and physical activity in early childhood to ensure children grow appropriately and develop healthy habits. Fifth, the comprehensive programs must promote healthy school environments, health and nutrition literacy, and physical activity among schoolage children and adolescents. Finally, it is important to provide family-based, multicomponent, lifestyle weight management services for children and young people who are obese.

Specifically for our study area, the federal government of Ethiopia should influence the food industry to lower the fat, sugar, and salt content of processed foods; guarantee that all consumers have access to and can afford healthy and nutritious options; limit the marketing of foods high in sugar, salt, and fat, particularly those targeted at children and teenagers; and promote regular physical activity in the workplace (44).

As a public health priority, the health sector of the town should concentrate on preventing overweight and obesity. Health officials should also work with the education sector of school district to mitigate problems among school-age adolescents by setting up play areas for physical activity and raising awareness through health professionals.

Adolescents should consume less sugar and fast food, eat more fruits, vegetables, and whole grains, and be physically active on a regular basis.

### Limitations

The shortcoming of the study was that it did not take genetic factors or parental BMI into account. Another problem could be recollection bias, which affects outcomes as participants may forget what they ate in the previous 7 days, and food portion size was not taken into account. In addition, both nutritional consumption and physical activity were self-reported data; therefore, overreporting or underreporting could be a significant issue in this study. Finally, only a sample of three chosen sub-cities was included in the study. Consequently, statistics from the omitted sub-cities may have produced a different result.

## Conclusion

Findings of this study revealed that the overall prevalence of overweight/obesity was 6.0%. Adolescents who are female, come from high-income families, and eat fast food frequently are more likely to be overweight or obese. Future health initiatives aimed at lowering the prevalence of overweight and obesity in adolescents should pay special attention to female adolescents and those from high-income families.

Additionally, more studies in this area should be conducted with a larger sample size, taking into account the portion size of food.

## Data Availability

The raw data supporting the conclusions of this article will be made available by the authors, without undue reservation.
